# Falls and EQ-5D rated quality of life in community-dwelling seniors with concurrent chronic diseases: a cross-sectional study

**DOI:** 10.1186/1477-7525-12-2

**Published:** 2014-01-08

**Authors:** Ulrich Thiem, Renate Klaaßen-Mielke, Ulrike Trampisch, Anna Moschny, Ludger Pientka, Timo Hinrichs

**Affiliations:** 1 Department of Geriatrics, Marienhospital Herne, University of Bochum, Widumer Str. 8, Herne D-44627, Germany; 2 Department of Medical Informatics, Biometry and Epidemiology, University of Bochum, Bochum, Germany; 3 Department of Sports Medicine and Sports Nutrition, University of Bochum, Bochum, Germany; 4 Swiss Paraplegic Research, Nottwil, Switzerland

**Keywords:** Falls, Accidental falls, Fear of falling, Multiple chronic diseases, Quality of life, EQ-5D, Elderly, Cross-sectional study

## Abstract

**Background:**

Although recommended for use in studies investigating falls in the elderly, the European Quality of Life Group instrument, EQ-5D, has not been widely used to assess the impact of falls on quality of life. The aim of this study was to investigate the association of single and frequent falls with EQ-5D rated quality of life in a sample of German community-dwelling seniors in primary care suffering a variety of concurrent chronic diseases and conditions.

**Methods:**

In a cross-sectional study, a sample of community-dwelling seniors aged ≥ 72 years was interviewed by means of a standardised telephone interview. According to the number of self-reported falls within twelve months prior to interview, participants were categorised into one of three fall categories: no fall vs. one fall vs. two or more falls within twelve months. EQ-5D values as well as other characteristics were compared across the fall categories. Adjustments for a variety of concurrent chronic diseases and conditions and further variables were made by using multiple linear regression analysis, with EQ-5D being the target variable.

**Results:**

In total, 1,792 participants (median age 77 years; 53% female) were analysed. The EQ-5D differed between fall categories. Participants reporting no fall had a mean EQ-5D score of 81.1 (standard deviation [s.d.]: 15.4, median: 78.3), while participants reporting one fall (n = 265; 14.8%) and participants with two or more falls (n = 117; 6.5%) had mean total scores of 77.0 (s.d.: 15.8, median: 78.3; mean difference to participants without a fall: -4.1, p < 0.05) and 72.1 (s.d.: 17.6, median: 72.5; mean difference: -9.0, p < 0.05), respectively. The mean difference between participants with one fall and participants with two or more falls was -4.9 (p < 0.05). Under adjustment for a variety of chronic diseases and conditions, the mean decrease in the total EQ-5D score was about -1.0 score point for one fall and about -2.5 points for two or more falls within twelve months. In quantity, this decrease is comparable to other chronic diseases adjusted for. Among the variables with the greatest negative association with EQ-5D ratings in multivariate analysis were depression and fear of falling.

**Conclusions:**

The findings suggest that falls are negatively associated with EQ-5D rated quality of life independent of a variety of chronic diseases and conditions.

## Introduction

Single and multiple falls are a special health concern in the elderly. According to recent European surveys, 20% and more of people aged ≥ 65 years suffer at least one fall within a year [1-3]. Falls are associated with several negative health consequences. Fall-related injury, especially fractures, are of concern [4,5], as are negative psycho-social consequences like reduced physical activity [6], fear of falling [7,8] and impairments in quality of life [8,9]. Recent health economic studies uncovered that falls are also associated with a considerable amount of health care costs [9,10].

Although the association between falls and quality of life has been widely accepted, the interrelationship is complex. It is known that people suffering at least one fall generally rate their quality of life lower than people without falls do [11-18]. Further, it is well established that the negative impact of falls is mediated at least in part by fear of falling [12,15,16,18-21]. However, the association of falls and impaired quality of life may also be confounded by some underlying deterioration that precedes falls and simultaneously impairs quality of life [11,22,23]. Further, studies investigating fall prevention interventions are inconclusive in terms of their ability to improve quality of life [24-31].

Along with the Health Survey Short Form 12 (SF-12) instrument, the ‘Prevention of Falls Network Europe’ (ProFaNE) consensus group recommended the use of the European Quality of Life Group instrument, the EQ-5D, for further studies [32]. The EQ-5D is easy to use and free of charge, when used for scientific purposes. Health utility indices are available that allow further health economic analyses based on EQ-5D ratings [33]. Despite this, only few studies used it in the context of falls and fall prevention [9,15,20,27]. One report demonstrated a strong negative influence of fear of falling on EQ-5D rated quality of life [15]. Unfortunately, the effect of other conditions in relation to fear of falling or falls was not investigated. Two of the studies investigated the impact of fall-related fractures, not falls alone, on the EQ-5D rated quality of life [9,20]. Finally, one study [27], a cluster-randomized controlled trial, reported the effects of an activity program in long term care facilities, but not EQ-5D values in relation to falls.

So far, the impact of falls on the EQ-5D rated quality of life has not been quantified in relation to other chronic diseases or health conditions, and data from German studies are lacking, too. Our aim, therefore, was to investigate the association of single and frequent falls with quality of life as rated with the EQ-5D in a sample of German community-dwelling seniors in primary care suffering a variety of concurrent chronic diseases and conditions.

## Methods

### Study sample

The cross-sectional sample used for this analysis was drawn during the seven year follow-up of a large cohort study initiated in 2001 with 6,880 participants from primary care practices throughout Germany. Details of the cohort are described elsewhere [34]. In brief, each of the participating 344 general practitioners consecutively recruited on average 20 eligible patients seeking primary health care during a predetermined week and fulfilling all inclusion criteria (age ≥ 65 years, being legally competent and able to cooperate appropriately, and providing written informed consent). The only exclusion criterion was life expectancy ≤ 6 months. The present study is part of a larger consortial research project [35].

During the seven year follow-up of the study cohort, telephone interviews were offered in addition to a clinical visit to all participants surviving until 2008 (n = 5,578). Of these, 3,445 could not be contacted by telephone or refused to take part. 196 people were successfully contacted, but were not able to perform a structured telephone interview.

The most common reasons for non-participation or inability to participate were: impaired overall health status, cognitive impairment, change of residency into a long-term care facility or nursing home, no interest in the issue studied by the telephone interview or no further interest to participate in the study at all. People finally taking part in the telephone interview (n = 1,937) were significantly younger, more often male than female, and had a higher overall educational level, as compared to non-participants. Further details have already been reported [36].

### Fall definition and fear of falling

Participants were interviewed by means of a standardised telephone interview. For the purpose of this study, we used the fall definition as proposed by the ProFaNE consensus group [32], which defines a fall as ‘an unexpected event in which the participant comes to rest on the ground, floor or lower level’. If an event fulfilling this definition was reported, the number of falls within the previous twelve months was recorded. Participants were categorised into one of three fall categories: no fall vs. one fall vs. two or more falls. To measure significant fall-related injury, the number of resulting fractures was noted. Fear of falling was assessed using a single categorical question, i.e. how much the participant was afraid of falling. Categories were ‘not at all’ , ‘only a bit’ , ‘being afraid’ , and ‘being much afraid’.

### Quality of life

For quality of life assessment, we used the EQ-5D [37,38]. The EQ-5D is a generic instrument to assess health related quality of life and contains five dimensions, i.e. mobility, self-care, usual activities, pain/discomfort and anxiety/depression. The person interviewed can choose between three answer categories for each of the domains in question, namely having no problem, having some problem or having extreme problems / being unable to perform an activity. This results in 243 (3^5^) theoretically possible combinations of answers. As recommended [37,38], we used population-based preference weights to assign single EQ-5D index scores to each combination. Raw values as used for our analysis can range from 0 to 100, with higher scores indicating better self-perceived health.

### Further measurements

Besides falls and quality of life, participants were further asked about a variety of chronic diseases and health conditions, i.e. hypertension, diabetes, coronary artery disease (CAD), chronic heart failure (CHF), peripheral artery disease (PAD), stroke history, Parkinson’s disease (PD), chronic obstructive pulmonary disease (COPD)/emphysema, arthritis, osteoporosis and/or chronic back pain/spinal disease. The question was always whether or not a physician ever made the diagnosis mentioned. Sporting activities were assessed by means of the PRISCUS-PAQ [39], walking ability by asking for the use of walking aids. The number of physician contacts within three months prior to interview was noted, as was the number of drugs currently taken on a regular basis. Depressive mood was measured by means of the Geriatric Depression Scale 15 (GDS-15) [40]. Pain during the past three months and sensory impairments, i.e. hearing loss or visual impairment, were also assessed. Participants’ sex, date of birth, and educational level were also documented.

### Statistical analysis

Categorical data are presented in absolute and relative frequencies, continuous variables with mean, standard deviation (s.d.) and median. Group differences across the three fall categories (no fall, one fall, two or more falls) were tested by means of a χ^2^-test for categorical variables. Mean EQ-5D scores were compared using analysis of variance (ANOVA) as a global test, and than pairwise using t-test. To quantify the association of different variables with the variable of interest, EQ-5D rated quality of life, we used multiple linear regression analysis. Acknowledging the multifactorial etiology of falls [41] and based on existing knowledge on potential risk factors for falls in community-dwelling elderly people [42-44], the following variables were included into the regression analyses: sex, age, education, hearing loss/visual impairment, sporting activities, physician contact within the past three months, fear of falling, depressive mood, chronic diseases (CAD, CHF, diabetes, hypertension, COPD/emphysema, stroke history, arthritis, PAD, Parkinson’s disease, osteoporosis/chronic back pain/spinal disease), number of drugs to take regularly, walking aid use, and pain within the past three months. The slope estimate for each explanatory variable is presented as well as p-values. Cases with missing values in one of the explanatory variables were excluded from analysis. Statistical significance was considered with p-values ≤ 0.05. All analyses were performed using SAS 9.1 software (SAS Institute Inc., Cary, NC, USA).

### Ethics approval

This study was performed in accordance with the Declaration of Helsinki of the World Medical Association [45]. The cohort study was approved by the institutional review board of the University of Heidelberg, Germany, and the University of Bochum, Germany. For the conduct of the cross-sectional study, a further approval was obtained from the institutional review board of the University of Bochum, Germany.

## Results

Of 1,937 people participating in the 7-year follow-up telephone interview, 145 participants were excluded because of incomplete data. In total, 1,792 participants aged ≥ 72 years were analysed. The mean age was 78.1 years (s.d.: 4.2 years, median: 77 years). 949 (53.0%) were female. Two hundred and sixty-five participants (14.8%) reported one fall within the previous twelve months. One hundred and seventeen participants (6.5%) suffered two or more falls in twelve months. Of 382 participants with at least one fall in twelve months, 54 (14.1%) reported a fracture as a fall-related injury. Characteristics of the participants for the total sample and for the different fall categories separately are depicted in Table 1.

**Table 1:** Characteristics of the total sample and by fall category (no fall vs. one fall vs. ≥ two falls) and group differences across the fall categories

**Variable**	**Total**		**Sample stratified by fall history**						
			**No fall**		**One fall**		**≥ two falls**		**χ**^**2**^**-test**
	**N = 1,792**		**N = 1,410**		**N = 265**		**N = 117**		
	**n**	**%**	**n**	%*	**n**	%*	**n**	%*	**p**
Female sex	949	53.0	702	49.8	176	66.4	71	60.7	< 0.001
Age ≥ 80 years	602	33.6	444	31.5	102	38.5	56	47.9	< 0.001
Low education	1,059	59.1	840	59.6	156	58.9	63	53.9	n.s.
Hypertension	1,160	64.7	895	63.5	180	67.9	85	72.7	n.s.
Diabetes	445	24.8	345	24.5	65	24.5	35	29.9	n.s.
CAD	457	25.5	358	25.4	65	24.5	34	29.1	n.s.
CHF	338	18.9	257	18.2	51	19.3	30	25.6	n.s.
PAD	214	11.9	166	11.8	30	11.3	18	15.4	n.s.
Stroke history	140	7.8	110	7.8	16	6.0	14	12.0	n.s.
COPD/emphysema	218	12.2	160	11.4	33	12.5	25	21.4	< 0.05
Arthritis	628	35.0	481	34.1	96	36.2	51	43.6	n.s.
Osteoporosis/chronic back pain/spinal disease	698	39.0	521	37.0	117	44.2	60	51.3	< 0.05
Parkinson’s disease	16	0.9	13	0.9	1	0.4	2	1.7	n.s.
Hearing loss/visual impairment	1,017	56.8	774	54.9	157	59.3	86	73.5	< 0.001
Depressive mood (GDS-15 ≥ 6)	142	7.9	93	6.6	26	9.8	23	19.7	< 0.001
Pain within past three months	950	53.0	731	51.8	146	55.1	73	62.4	n.s.
Sporting activities >1 h / week	1,036	57.8	821	58.2	145	54.7	70	59.8	n.s.
Walking aid use	299	16.7	192	13.6	73	27.6	34	29.1	< 0.001
Physician contact within the past three months > 3	1,216	67.9	945	67.0	184	69.4	87	74.4	n.s.
Drugs to take regularly ≥ 6	909	50.7	708	50.2	136	51.3	65	55.6	n.s.
Severe fear of falling	143	8.0	85	6.0	39	14.7	19	16.2	< 0.001

*The given percentages refer to the respective subsample.

CAD: coronary artery disease; CHF: chronic heart failure; PAD: peripheral artery disease; COPD: chronic obstructive pulmonary disease; GDS: Geriatric Depression Scale; n. s.: not significant (i.e. p > 0.05).

The mean total score of the EQ-5D was 79.9 (s.d.: 15.8, median: 78.3) for the whole sample. The EQ-5D differed between fall categories. Participants without falls within twelve months prior to interview had a mean EQ-5D score of 81.1 (s.d.: 15.4, median: 78.3). By contrast, participants reporting one fall and participants with two or more falls had mean total scores of 77.0 (s.d.: 15.8, median: 78.3; mean difference to participants without a fall: -4.1, p < 0.05) and 72.1 (s.d.: 17.6, median: 72.5; mean difference to participants without a fall: -9.0, p < 0.05), respectively. The mean difference between participants with one fall and participants with two or more falls was -4.9 (p < 0.05).

Multiple linear regression analysis revealed a negative relationship between falls and EQ-5D rated quality of life. Under adjustment for a variety of chronic diseases and conditions, the mean decrease in the total EQ-5D score was about -2.5 points for two or more falls within twelve months. In terms of its quantity, this decrease is comparable to other chronic diseases assessed and adjusted for, as can be seen from Table 2. As could be expected, depressive mood and fear of falling were among the variables with the strongest negative association with quality of life. However, even under adjustment for these variables frequent falling retained an independent negative association with quality of life in this analysis.

**Table 2:** Results of the multivariate linear regression analysis: associations of fall categories, fear of falling, depressive mood, chronic diseases and conditions with EQ-5D rated quality of life (N = 1,792)

**Variable**	**Estimate**	**p-value**
One fall	−1.05	n. s.
Two or more falls	−2.50	< 0.05
Fear of falling	−7.30	< 0.001
Depressive mood (GDS-15)	−13.13	< 0.001
CAD	0.76	n. s.
CHF	0.08	n. s.
Diabetes	−0.10	n. s.
Hypertension	−0.24	n. s.
COPD/emphysema	−2.59	< 0.01
Stroke history	−2.86	< 0.01
Arthritis	−3.24	< 0.001
PAD	−3.44	< 0.001
Parkinson’s disease	−3.52	n. s.
Osteoporosis/chronic back pain/spinal disease	−3.87	< 0.001
Drugs to take regularly ≥ 6	−2.03	< 0.01
Walking aid use	−6.52	< 0.001
Pain within the past three months	−9.67	< 0.001

Multiple linear regression model adjusted for all variables in the table and additionally for: sex, age, education, hearing loss / visual impairment, sporting activities, physician contact within the past three months. n. s.: not significant (i.e. p > 0.05); GDS: Geriatric Depression Scale; CAD: coronary artery disease; CHF: chronic heart failure; COPD: chronic obstructive pulmonary disease; PAD: peripheral artery disease.

## Discussion

Our data show that single and frequent falls, a common health problem in community-dwelling seniors, are negatively associated with quality of life, as rated with the EQ-5D in this study. In the univariate analysis, the mean differences in total EQ-5D scores were -4.1 and -9.0 for single and frequent falls, respectively. Although adjusted for a large number of covariates and potential confounders, including fear of falling and depressive mood, frequent falls retained a statistically significant association to quality of life in multivariate analysis. The mean adjusted decrease in EQ-5D score of -2.5 for frequent falls was comparable to other chronic diseases and conditions, like COPD or stroke history. With this, our data suggest that falls may have a clinically meaningful impact on people affected and deserve the same attention in terms of quality of life as some established chronic diseases do.

Our findings are in line with several other studies. In an analysis of three different datasets from two randomized controlled trials and one cohort study, a marked impact of fractures, fear of falling and falls on the EQ-5D rated quality of life was found [15]. A fracture resulting from a fall had an impact twice as large as a fall, as could be expected. Comparable findings in terms of fractures of different sites were recently reported in a large trauma sample from the Netherlands [9], which also used the EQ-5D to assess quality of life. In a recent Brazilian survey among elderly aged ≥ 60 years, impairments of quality of life as assessed by the Health Survey Short Form 36 (SF-36) instrument were closely related to falls [13]. Of note, this association remained independent after adjustment for GDS-15 rated depression, comparable to our findings. Unfortunately, none of the studies mentioned investigated the role of other concurrent chronic diseases in the context of falling by multivariate analysis.

In a recent analysis of a larger insurance company sample from the United States, impairments of quality of life due to falls and fall risk were described as being comparable in quantity to most other chronic diseases present [14]. This report supports our findings. However, quality of life was not assessed with the EQ-5D, and the models used for adjustments were targeting falls as the variable of interest, not quality of life, which makes comparisons difficult. In another recent analysis [46], the effect of falls as well as some other chronic conditions including diabetes, mobility impairment and pain was dependent on whether or not depression was present.

Apart from the studies mentioned, several earlier reports already described the association between either falls or fractures resulting from falls and quality of life, using a variety of different quality of life measures [16-18,20,31]. Our findings are well in line with these reports.

Several limitations of our study should be considered. At first, our results are only applicable to community-dwelling seniors with relatively good overall health status. The sample of participants contained younger and better educated people, and mostly people living independently at home. By contrast, change of residency to a nursing home, impairments in overall health status, cognitive impairment and overt dementia were all causes for non-participation. As all these conditions are related to both the frequency of falling and quality of life, our conclusions cannot be transferred to this patient group. Secondly, we cannot exclude residual confounding in our analysis. Fear of falling was not assessed by means of a standard instrument, e.g. the ‘Falls Efficacy Scale – International’ (FES-I) [47], but simply by a single, categorical question. Thus, it is possible that the phenomenon of fear of falling was only incompletely assessed. The same applies to depression that was only assessed by means of a screening tool, the GDS-15. However, our intention was not to investigate the mechanism by which falls may influence quality of life. Our aim was to demonstrate the association of falls with quality of life accounting for variables readily available in primary care. Detailed information about fear of falling and depression are usually not available when a patient is reporting a fall. Rather, investigations in mood and fear may be prompted by falls. Our analysis already adjusted for fear of falling and depression, although perhaps incomplete. However, as the analysis still reveals an independent association of falls with quality of life, this should even more apply to a clinical situation in which fear of falling and depression are not accounted for easily. Thirdly, by design, we can only describe associations, not establish causality. There is evidence that reduced quality of life may precede falls [22,23]. If so, falls would be the result of some other alteration that also impairs quality of life, for example more advanced functional deficits in domains like cognition or activities of daily living. With our data, however, we have no chance to investigate this interesting issue further.

Despite these limitations, we believe that our findings are important for others. Researchers may find the EQ-5D measures useful for further study planning and sample size estimation. Clinicians and care givers interested in the quality of life of elderly people will feel encouraged to account for falls, as the strength of their association with quality of life appears to be comparable to that of other well established and recognised chronic diseases and conditions.

## Conclusions

Our study shows that falls are associated with impairments in the EQ-5D rated quality of life. Although a variety of other diseases and conditions were considered, falls kept an independent negative association to quality of life in our analysis. This implies that as far as quality of life is concerned, falls deserve the same attention as some other well established and recognised diseases and conditions do.

## Competing interests

This work was supported by an unrestricted educational grant by Sanofi-Aventis, Berlin, Germany (2001–2007), and the German Federal Ministry of Education and Research (‘Bundesministerium für Bildung und Forschung’, BMBF, grant no. 01ET0720, since 2007). The study was conducted within the PRISCUS research consortium (‘Prerequisites for a new health care model for elderly people with multimorbidity’).

## Authors’ contributions

UTh, LP and TH obtained funding; UTh, UTr, AM, LP and TH designed the study; UTh, RKM and UTr supervised data acquisition and preparation; RKM and UTh performed the analysis; UTh, RKM, UTr, AM and TH interpreted the results; UTh drafted the manuscript; all authors revised the manuscript draft critically for important intellectual content; all authors read and approved the final version of the manuscript.

## References

[B1] Olsson Moller U Midlov P Kristensson J Ekdahl C Berglund J Jakobsson U Prevalence and predictors of falls and dizziness in people younger and older than 80 years of age-A longitudinal cohort study Arch Gerontol Geriatr 2013 56 160 168 10.1016/j.archger.2012.08.013 22999306

[B2] Skalska A Wizner B Piotrowicz K Klich Raczka A Klimek E Mossakowska M Rowinski R Kozak Szkopek E Jozwiak A Gasowski J Grodzicki T The prevalence of falls and their relation to visual and hearing impairments among a nation-wide cohort of older Poles Exp Gerontol 2013 48 2 140 146 10.1016/j.exger.2012.12.003 23261517

[B3] Schumacher J Pientka L Trampisch U Moschny A Hinrichs T Thiem U The prevalence of falls in adults aged 40 years or older in an urban, German population: results from a telephone survey Z Gerontol Geriatr 2013. PMID: 23743881 [Epub ahead of print] 10.1007/s00391-013-0503-y 23743881

[B4] Balzer K Bremer M Schramm S Lühmann D Raspe H Falls prevention for the elderly GMS Health Technol Assess 2012 8 Doc01 22536299 10.3205/hta000099 PMC3334922

[B5] Bischoff-Ferrari HA The role of falls in fracture prediction Curr Osteoporos Rep 2011 9 116 121 10.1007/s11914-011-0059-y 21655932

[B6] Gregg EW Pereira MA Caspersen CJ Physical activity, falls, and fractures among older adults: a review of the epidemiologic evidence J Am Geriatr Soc 2000 48 883 893 10968291 10.1111/j.1532-5415.2000.tb06884.x

[B7] Scheffer AC Schuurmans MJ van Dijk N van der Hooft T de Rooij SE Fear of falling: measurement strategy, prevalence, risk factors and consequences among older persons Age Ageing 2008 37 19 24 18194967 10.1093/ageing/afm169

[B8] Visschedijk J Achterberg W Van Balen R Hertogh C Fear of falling after hip fracture: a systematic review of measurement instruments, prevalence, interventions, and related factors J Am Geriatr Soc 2011 58 1739 1748 20863333 10.1111/j.1532-5415.2010.03036.x

[B9] Hartholt KA van Beeck EF Polinder S van der Velde N van Lieshout EM Panneman MJ van der Cammen TJ Patka P Societal consequences of falls in the older population: injuries, healthcare costs, and long-term reduced quality of life J Trauma 2011 71 748 753 10.1097/TA.0b013e3181f6f5e5 21045738

[B10] Heinrich S Rapp K Rissmann U Becker C Konig HH Cost of falls in old age: a systematic review Osteoporos Int 2010 21 891 902 10.1007/s00198-009-1100-1 19924496

[B11] Bilotta C Bowling A Nicolini P Case A Pina G Rossi SV Vergani C Older People's Quality of Life (OPQOL) scores and adverse health outcomes at a one-year follow-up. A prospective cohort study on older outpatients living in the community in Italy Health Qual Life Outcomes 2011 9 72 10.1186/1477-7525-9-72 21892954 PMC3175435

[B12] Chang NT Chi LY Yang NP Chou P The impact of falls and fear of falling on health-related quality of life in Taiwanese elderly J Community Health Nurs 2010 27 84 95 10.1080/07370011003704958 20437289

[B13] Coimbra AM Ricci NA Coimbra IB Costallat LT Falls in the elderly of the family health program Arch Gerontol Geriatr 2010 51 317 322 10.1016/j.archger.2010.01.010 20153535

[B14] Hawkins K Musich S Ozminkowski RJ Bai M Migliori RJ Yeh CS The burden of falling on the quality of life of adults with Medicare supplement insurance J Gerontol Nurs 2011 37 36 47 21485987 10.3928/00989134-20110329-03

[B15] Iglesias CP Manca A Torgerson DJ The health-related quality of life and cost implications of falls in elderly women Osteoporos Int 2009 20 869 878 10.1007/s00198-008-0753-5 18846400

[B16] Ozcan A Donat H Gelecek N Ozdirenc M Karadibak D The relationship between risk factors for falling and the quality of life in older adults BMC Public Health 2005 5 90 10.1186/1471-2458-5-90 16124871 PMC1208910

[B17] Roe B Howell F Riniotis K Beech R Crome P Ong BN Older people and falls: health status, quality of life, lifestyle, care networks, prevention and views on service use following a recent fall J Clin Nurs 2009 18 2261 2272 10.1111/j.1365-2702.2008.02747.x 19583659

[B18] Suzuki M Ohyama N Yamada K Kanamori M The relationship between fear of falling, activities of daily living and quality of life among elderly individuals Nurs Health Sci 2002 4 155 161 10.1046/j.1442-2018.2002.00123.x 12406202

[B19] Li F Fisher KJ Harmer P McAuley E Wilson NL Fear of falling in elderly persons: association with falls, functional ability, and quality of life J Gerontol B Psychol Sci Soc Sci 2003 58 P283 290 10.1093/geronb/58.5.P283 14507935

[B20] Salkeld G Cameron ID Cumming RG Easter S Seymour J Kurrle SE Quine S Quality of life related to fear of falling and hip fracture in older women: a time trade off study BMJ 2000 320 341 346 10.1136/bmj.320.7231.341 10657327 PMC27279

[B21] Warnke A Meyer G Bott U Muhlhauser I Validation of a quality of life questionnaire measuring the subjective fear of falling in nursing home residents Z Gerontol Geriatr 2004 37 459 466 10.1007/s00391-004-0214-5 15614598

[B22] Rohde G Haugeberg G Mengshoel AM Moum T Wahl AK Is global quality of life reduced before fracture in patients with low-energy wrist or hip fracture? A comparison with matched controls Health Qual Life Outcomes 2008 6 90 10.1186/1477-7525-6-90 18980668 PMC2613869

[B23] Rohde G Mengshoel AM Wahl AK Moum T Haugeberg G Is health-related quality of life associated with the risk of low-energy wrist fracture: a case-control study BMC Musculoskelet Disord 2009 10 80 10.1186/1471-2474-10-80 19573252 PMC2714004

[B24] Cakar E Dincer U Kiralp MZ Cakar DB Durmus O Kilac H Soydan FC Sevinc S Alper C Jumping combined exercise programs reduce fall risk and improve balance and life quality of elderly people who live in a long-term care facility Eur J Phys Rehabil Med 2010 46 59 67 20332728

[B25] de Vries OJ Peeters GM Elders PJ Muller M Knol DL Danner SA Bouter LM Lips P Multifactorial intervention to reduce falls in older people at high risk of recurrent falls: a randomized controlled trial Arch Intern Med 2010 170 1110 1117 20625015 10.1001/archinternmed.2010.169

[B26] Kerse N Hayman KJ Moyes SA Peri K Robinson E Dowell A Kolt GS Elley CR Hatcher S Kiata L Home-based activity program for older people with depressive symptoms: DeLLITE–a randomized controlled trial Ann Fam Med 2010 8 214 223 10.1370/afm.1093 20458104 PMC2866718

[B27] Kerse N Peri K Robinson E Wilkinson T von Randow M Kiata L Parsons J Latham N Parsons M Willingale J Does a functional activity programme improve function, quality of life, and falls for residents in long term care? Cluster randomised controlled trial BMJ 2008 337 a1445 10.1136/bmj.a1445 18845605 PMC2565754

[B28] Sjosten NM Vahlberg TJ Kivela SL The effects of multifactorial fall prevention on depressive symptoms among the aged at increased risk of falling Int J Geriatr Psychiatry 2008 23 504 510 10.1002/gps.1927 17932996

[B29] Smulders E Weerdesteyn V Groen BE Duysens J Eijsbouts A Laan R van Lankveld W Efficacy of a short multidisciplinary falls prevention program for elderly persons with osteoporosis and a fall history: a randomized controlled trial Arch Phys Med Rehabil 2010 91 1705 1711 10.1016/j.apmr.2010.08.004 21044715

[B30] Szanton SL Thorpe RJ Boyd C Tanner EK Leff B Agree E Xue QL Allen JK Seplaki CL Weiss CO Community aging in place, advancing better living for elders: a bio-behavioral-environmental intervention to improve function and health-related quality of life in disabled older adults J Am Geriatr Soc 2012 59 2314 2320 22091738 10.1111/j.1532-5415.2011.03698.x PMC3245364

[B31] Vaapio SS Salminen MJ Ojanlatva A Kivela SL Quality of life as an outcome of fall prevention interventions among the aged: a systematic review Eur J Public Health 2009 19 7 15 18971207 10.1093/eurpub/ckn099

[B32] Lamb SE Jorstad-Stein EC Hauer K Becker C Development of a common outcome data set for fall injury prevention trials: the Prevention of Falls Network Europe consensus J Am Geriatr Soc 2005 53 1618 1622 10.1111/j.1532-5415.2005.53455.x 16137297

[B33] Dolan P Gudex C Kind P Williams A A Social Tariff for EuroQoL: Results from a UK General Population Survey 1995 University of York, UK: Centre for Health Economics Discussion Paper 138

[B34] Diehm C Allenberg JR Pittrow D Mahn M Tepohl G Haberl RL Darius H Burghaus I Trampisch HJ Mortality and vascular morbidity in older adults with asymptomatic versus symptomatic peripheral artery disease Circulation 2009 120 2053 2061 10.1161/CIRCULATIONAHA.109.865600 19901192

[B35] Thiem U Theile G Junius-Walker U Holt S Thurmann P Hinrichs T Platen P Diederichs C Berger K Hodek JM Prerequisites for a new health care model for elderly people with multimorbidity: the PRISCUS research consortium Z Gerontol Geriatr 2011 44 115 120 10.1007/s00391-010-0156-z 21161244

[B36] Hinrichs T Moschny A Klaassen-Mielke R Trampisch U Thiem U Platen P General practitioner advice on physical activity: analyses in a cohort of older primary health care patients (getABI) BMC Fam Pract 2011 12 26 10.1186/1471-2296-12-26 21569227 PMC3115873

[B37] Szende A Oppe M Devlin N EQ-5D value sets - inventory, comparative review and user guide 2007 Heidelberg, Germany: Springer

[B38] Greiner W Weijnen T Nieuwenhuizen M Oppe S Badia X Busschbach J Buxton M Dolan P Kind P Krabbe P A single European currency for EQ-5D health states. Results from a six-country study Eur J Health Econ 2003 4 222 231 10.1007/s10198-003-0182-5 15609189

[B39] Trampisch U Platen P Burghaus I Moschny A Wilm S Thiem U Hinrichs T Reliability of the PRISCUS-PAQ. Questionnaire to assess physical activity of persons aged 70 years and older Z Gerontol Geriatr 2010 43 399 406 10.1007/s00391-010-0118-5 20967452

[B40] Burke WJ Roccaforte WH Wengel SP Conley DM Potter JF The reliability and validity of the geriatric depression rating scale administered by telephone J Am Geriatr Soc 1995 43 674 679 7775729 10.1111/j.1532-5415.1995.tb07205.x

[B41] American Geriatrics Society, British Geriatrics Society, and American Academy of Orthopaedic Surgeons Panel on Falls Prevention Guideline for the prevention of falls in older persons J Am Geriatr Soc 2001 49 664 672 10.1046/j.1532-5415.2001.49115.x 11380764

[B42] Deandrea S Lucenteforte E Bravi F Foschi R La Vecchia C Negri E Risk factors for falls in community-dwelling older people: a systematic review and meta-analysis Epidemiology 2010 21 658 668 10.1097/EDE.0b013e3181e89905 20585256

[B43] Kvelde T McVeigh C Toson B Greenaway M Lord SR Delbaere K Close JC Depressive symptomatology as a risk factor for falls in older people: systematic review and meta-analysis J Am Geriatr Soc 2013 61 694 706 10.1111/jgs.12209 23617614

[B44] Bloch F Thibaud M Dugue B Breque C Rigaud AS Kemoun G Episodes of falling among elderly people: a systematic review and meta-analysis of social and demographic pre-disposing characteristics Clinics (Sao Paulo) 2010 65 895 903 10.1590/S1807-59322010000900013 21049218 PMC2954741

[B45] World Medical Association (WMA) Declaration of Helsinki: Ethical Principles for Medical Research Involving Human Subjects 2004 Tokyo, Japan: 55th WMA General Assembly

[B46] Ozminkowski RJ Musich S Bottone FG Jr Hawkins K Bai M Unutzer J Hommer CE Migliori RJ Yeh CS The burden of depressive symptoms and various chronic conditions and health concerns on the quality of life among those with Medicare Supplement Insurance Int J Geriatr Psychiatry 2012 27 948 958 10.1002/gps.2806 22025352

[B47] Dias N Kempen GI Todd CJ Beyer N Freiberger E Piot-Ziegler C Yardley L Hauer K [The German version of the Falls Efficacy Scale-International Version (FES-I)] Z Gerontol Geriatr 2006 39 297 300 10.1007/s00391-006-0400-8 16900450

